# COVID-19 and Lung Cancer Survival: An Updated Systematic Review and Meta-Analysis

**DOI:** 10.3390/cancers14225706

**Published:** 2022-11-21

**Authors:** Simone Oldani, Fausto Petrelli, Giuseppina Dognini, Karen Borgonovo, Maria Chiara Parati, Mara Ghilardi, Lorenzo Dottorini, Mary Cabiddu, Andrea Luciani

**Affiliations:** Oncology Unit, ASST Bergamo Ovest, 24047 Treviglio (BG), Italy

**Keywords:** COVID-19, lung cancer, risk, mortality, meta-analysis

## Abstract

**Simple Summary:**

The coronavirus disease (COVID-19) pandemic has increased morbidity and mortality in the general population. Patients with cancer have immune dysfunction due to the use of immunosuppressive agents, poor nutritional status, or the direct effects of the tumor on the reactivity of the immune system, making oncological patients more susceptible to severe disease and death. In this systematic review and meta-analysis, we evaluated whether lung cancer increases the risk of severe COVID-19 and the risk of dying from the disease. We found that mortality in patients with lung cancer was significantly higher than that in control patients (HR = 2.00 [95%CI 1.52, 2.63], *p* < 0.01) or with other malignancies (HR = 1.91 [95%CI 1.53, 2.39], *p* < 0.01). In addition, we also observed a higher risk of severe infection in patients with lung cancer (HR = 1.47 [95%CI 1.06, 2.03], *p* = 0.02). We suggest that, in this setting, vaccine use may be considered a useful and mandatory measure to save lives.

**Abstract:**

Introduction: The outbreak of COVID-19 poses an unprecedented challenge to global public health. Patients with cancer are at a higher risk during the SARS-CoV-2 pandemic. Patients with lung cancer and COVID-19 were compared to those without cancer and those with other malignancies for the main outcome of this study. The aim of this study was to evaluate the differences in susceptibility, disease severity, and mortality between lung cancer patients and the general population. Methods: Using PRISMA reporting guidelines, we conducted a systematic review and meta-analysis of the published literature. The Cochrane Library database, PubMed, EMBASE, and PubMed Central were comprehensively searched for published papers until 31 May 2022. A pooled risk ratio (OR) with 95% CI was presented as the result of this meta-analysis. Results: We included 29 studies involved 21,257 patients with lung cancer and SARS-CoV-2 infection. Analysis data showed that mortality in patients with lung cancer was significantly higher than that in patients without cancer (HR = 2.00 [95%CI 1.52, 2.63], *p* < 0.01) or with other malignancies (HR = 1.91 [95%CI 1.53, 2.39], *p* < 0.01). In addition, we also observed a higher risk of severe infection in terms of life-threatening or required ICU admission/mechanical ventilation for lung cancer patients (HR = 1.47 [95%CI 1.06, 2.03], *p* = 0.02) than for patients with no cancer or other malignancies. Regarding lung cancer as a risk factor for acquiring SARS-CoV-2 infection, we could not reach statistical significance (hazard ratio [HR] =2.73 [95%CI 0.84, 8.94], *p* = 0.1). Conclusion: Lung cancer represents an important comorbidity and modifies COVID-19 prognosis in terms of disease severity and mortality. More patients experience severe or even fatal events. Considering their inherent fragility, patients with lung cancer, and generally all oncological populations, should be treated more carefully during the COVID-19 pandemic.

## 1. Introduction

The outbreak of a global pandemic due to coronavirus disease 2019 (COVID-19), which is caused by a novel severe acute respiratory syndrome coronavirus 2 (SARS-CoV-2), represents an unprecedented crisis in global public health. Hospital mortality rate is estimated approximately at 15–20% but increases to 40% in the cohort of patients who require ICU admission [1]. The clinical spectrum of COVID-19 ranges from asymptomatic infection to severe respiratory failure and death [2].

Cancer patients comprise a heterogeneous population because they have various risk factors for the development of clinical complications and severe disease due to coagulation disorders (hypercoagulability), immunological impairment, and immunosuppressive curative/palliative treatments [3,4].

Despite the accumulation of real-world data and evidence, the effect of cancer on COVID-19 outcomes has not been fully characterized. Numerous meta-analyses have evaluated mortality in cancer patients with COVID-19 to obtain accurate mortality risk estimates, but these have potential bias in study selection and data aggregation, such as the lack of adjusted survival estimates, non-cancer controls, or laboratory-confirmed infection as inclusion criteria. However, all these studies demonstrated an increased mortality rate in cancer patients who acquired SARS-CoV-2 infection.

In this study, we performed a systematic review and meta-analysis of the risks of COVID-19, severe disease, and mortality in patients with lung cancer. The lung is also a principal target of SARS-CoV-2. Evidence proves that SARS-CoV-2 enters the host cell through the SARS-CoV receptor, ACE2. A clinically tested inhibitor of the cellular serine protease TMPRSS2, which is involved in the priming of the SARS-CoV-2 S protein, can block viral penetration into the cell. SARS-CoV infects pneumocytes and macrophages in lungs [5].

However, SARS-CoV-2 spread has been observed in the extrapulmonary region because ACE2 is not only expressed in the lungs [6].

Nadalin et al. explored the association between nicotine dependence and the insertion/deletion (I/D) polymorphism of ACE in patients with lung cancer and demonstrated a correlation between this type of polymorphism and the risk of nicotine dependence and smoking severity in patients with lung cancer [7]. However, another review showed that ACE I/D polymorphism is not associated with the risk of lung cancer [8].

Therefore, there may be no common target among diseases; however, patients with cancer commonly have immune dysfunction due to the use of immunosuppressive agents, poor nutritional status, or direct effects of the tumor on the reactivity of the immune system [9]. This makes oncological patients more susceptible to severe respiratory diseases and more likely to die.

This study aimed to determine whether lung cancer is a poor prognostic factor for COVID-19.

## 2. Material and Methods

The systematic review followed the recommendations of the Preferred Reporting Items for Systematic Reviews and Meta-Analyses (PRISMA). The protocol has not been registered.

### 2.1. Search Strategy and Literature Search

PubMed, EMBASE, and The Cochrane Library databases were searched for articles published until 31 May 2022. Duplicate publications were identified and removed from the references of these articles. The search strategy included the following terms: (“lung cancer” or “lung carcinoma”) and (COVID-19).

### 2.2. Study Selection

All titles and abstracts were independently screened by two authors (FP and SO) after the initial search. The inclusion criteria were any retrospective, case-control, or cohort study with or without a control group (defined as patients without lung cancer but with COVID-19 infection) that (1) was published in English, (2) included patients with lung cancer and confirmed or suspected COVID-19 infection, and (3) reported outcome (mortality) and/or severity compared with non-cancer patients or no lung cancer patients reported.

In addition, studies were excluded if they had fewer than 10 patients, conference papers, abstracts, or preprints; if full-text articles could not be retrieved; and if they involved animals or children. The authors selected the studies with the largest and most updated cohorts among those reporting an overlapping series. Discrepancies were resolved through consensus.

### 2.3. Data Extraction and Quality Assessment

Two of us (FP and SO) independently extracted the following data: first author, study type, country of data collection, median follow up, patient’s treatment and stage, and unadjusted and adjusted odds ratios (ORs) or risk ratios (RRs) for severe disease and death for lung cancer patients. The quality of the included studies was assessed using the Newcastle–Ottawa scale. Publication bias across studies was assessed using the ROBINS tool for nonrandomized studies.

### 2.4. Outcomes and Statistical Analysis

The main outcome of interest was survival in patients with lung cancer and COVID-19. We compared survival in patients with lung cancer and COVID-19 infection with (1) control patients with no cancer and COVID-19 infection, and (2) patients with other cancers and COVID-19 infection. The results of this meta-analysis were presented as pooled risk ratios (ORs) with 95% confidence intervals (CIs).

Additionally, we conducted a meta-analysis of infection severity in patients with and without lung cancer. Patients with lung cancer were also assessed for infection risk.

Between-study statistical heterogeneity was quantified according to the random-effects heterogeneity parameter tau, and I2 statistics (the percentage of variance in effect estimates due to statistical heterogeneity rather than sampling error) were calculated for all meta-analyses.

To estimate between-study heterogeneity, all meta-analyses were conducted using a random-effects model, with limited maximum likelihood to account for statistical heterogeneity due to variability in study design and participant characteristics. The I2 statistic was used to quantify statistical heterogeneity.

Meta-analyses were performed with the RevMan software. A 2-sided *p* < 0.05 indicated statistical significance.

## 3. Results

**Figure 1:**
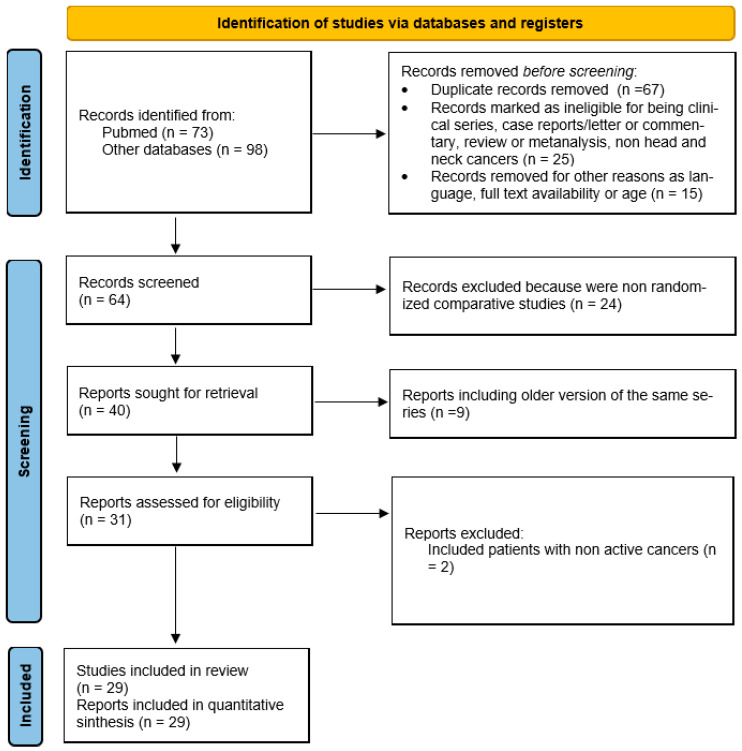
Flow diagram of included studies.

The initial search retrieved 171 articles for review (Figure 1). After the inclusion of records that were identified through additional sources and the removal of duplicate articles, a total of 29 studies [10,11,12,13,14,15,16,17,18,19,20,21,22,23,24,25,26,27,28,29,30,31,32,33,34,35,36,37,38] were included in this systematic review and meta-analysis. Except for the series by Wang et al., which included patients who received anti-COVID vaccines, all other series had their observation period in the pre-vaccine months. All patients had active cancers and were receiving or had recently received active anti-neoplastic treatments (either systemic therapies alone or with radiotherapy). Stage was not specified or was reported for all cancer types. All studies included lung cancer patients except in 2 studies where all thoracic malignancies were observed. However, in these studies, only a minority had mesothelioma (4 and 6%, respectively).

### 3.1. Characteristics of Included Studies

The 29 studies involved 21,257 patients with lung cancer and SARS-CoV-2 infection (Table 1; Figure 1) and consisted of 6 prospective, 1 cohort, 2 retrospective/prospective studies, 1 cross-sectional study, and 19 retrospective studies. In terms of study origin, 5 were from Asia, 9 from America (including Brazil), 14 from Europe, and 1 was a multicentric international study.

### 3.2. Infection Risk of Patients with Lung Cancer vs. Control Patients

**Figure 2:**
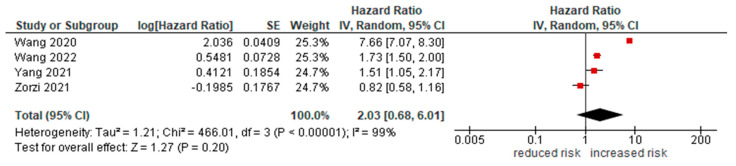
Forest plot for risk of infection in lung cancer patients.

Only 4 studies [34,35,36,38] reported this data. Overall, a diagnosis of lung cancer increased the risk of COVID-19 infection (HR = 2.73 [95%CI 0.84, 8.94], *p* = 0.1; Figure 2).

### 3.3. Severity of COVID-19 with Lung Cancer

**Figure 3:**
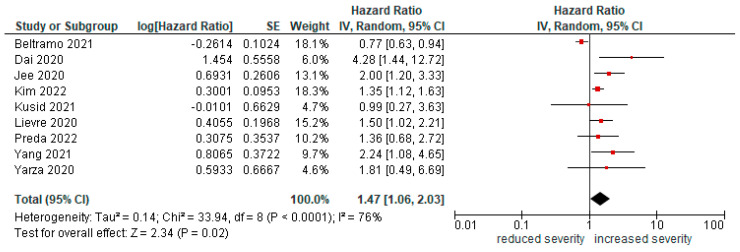
Forest plot for risk of severe infection in lung cancer patients.

Nine studies [13,17,23,24,25,27,31,36,37] reported the risk of severe infection (life-threatening or requiring ICU admission/mechanical ventilation) in patients with lung cancer compared to non-lung cancer patients. Lung cancer diagnosis increased the risk of severe disease (HR = 1.47 [95%CI1.06, 2.03], *p* = 0.02; Figure 3). Compared with other cancers, severity is increased (HR = 1.39 [1, 1.93], *p* = 0.05).

### 3.4. Mortality of Patients with Lung Cancer vs. Control Patients

**Figure 4:**
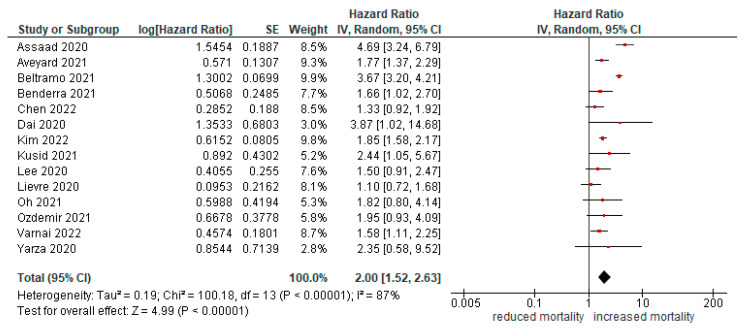
Forest plot for risk of mortality in lung cancer patients.

Fourteen papers [10,11,13,14,16,17,24,25,26,27,29,30,33,37] reported the risk of mortality in patients with respiratory or lung cancers. The risk of death was double in patients with cancer compared with those without cancer (HR = 2.00 [95%CI1.52, 2.63], *p* < 0.01; Figure 4). All studies reported HR adjusted for multivariate analysis except for the study by Assad et al. After exclusion of this study, risk of death remained significant even in multivariate analysis (HR = 1.85 [95%CI 1.4, 2.44], *p* < 0.01).

### 3.5. Mortality of Patients with Lung Cancer vs. Non Lung Cancer Patients

**Figure 5:**
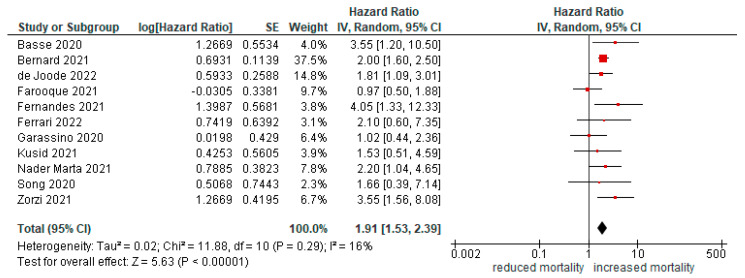
Forest plot for risk of mortality in lung cancer compared with other malignancies.

In patients with lung cancer, mortality due to COVID-19 infection was also higher than that due to other cancers (HR = 1.91 [95%CI 1.53, 2.39], *p* < 0.01; Figure 5).

## 4. Discussion

In this study, we present a systematic review of the literature on the link between lung cancer and COVID-19 in terms of susceptibility, severity of disease, and mortality. We achieved a large sample size, including 29 studies involving 21,257 patients from a wide range of countries. We found that lung cancer was an independent prognostic factor for survival in COVID-19 infected patients. Although many subgroups of lung cancer were included in these series, all studies pooled them together and did not split outcomes according to histology (non-small cell vs. small-cell lung cancers).

According to two previous meta-analyses on SARS-CoV-2 infection and cancer [39,40], lung neoplasm is associated with an increased risk of severe COVID-19 or death compared with control patients without infection. However, we expanded the published evidence and increased the number of included studies, reaching more than 20,000 cases of lung cancer patients.

Poor prognosis could be explained by chronic compromised clinical status, comorbidities (for example, respiratory disease in active or former smokers), immunocompromised state caused by cancer or its therapies, supportive medications such as steroids, and malnutrition. We do not know whether lung cancer enhances susceptibility to SARS-CoV-2, and we cannot reach statistical significance in this study. It will be interesting to define whether it represents an independent risk factor or if increased susceptibility is determined by common risk factors (smoking history, pollution, and chronic pulmonary disease).

Several studies have observed that oncological patients who recently received cancer treatment, such as chemotherapy, targeted therapy, or immunotherapy, had significantly higher COVID-19-related mortality rates than those who did not receive recent treatment [41]. These data demonstrate a role of oncological treatment in SARS-CoV-2 mortality. Unfortunately, the study included in this meta-analysis did not specify whether patients were in active treatment or not and when they received the last dose of treatment.

A study published in Seminars in Oncology in 2020 [42] demonstrated that the incidence of SARS-CoV-2 infection in patients affected by melanoma after treatment with immune-checkpoint inhibitors was lower than that in the overall Italian population as a control. Therefore, immunotherapy seems to have a positive effect and should be continued without delay in most patients, reserving precautionary delay only in frail people.

In addition to the acute morbidity and mortality from COVID-19, it is important to highlight that recent evidence shows reduced access to oncological treatments due to the long-term consequences of COVID-19, which affects approximately 15% of cancer patients [43].

The emergency horizon linked to the rapid spread of SARS-CoV-2 is wider than the complications related to viral infections in patients with cancer.

The SARS-CoV-2 pandemic involved all levels of cancer care, including screening, diagnosis, and active oncological treatment [44]. According to data from other cancers, in 2020, there was a drop in new lung neoplasm diagnosis [45,46]: undiagnosed cancer diseases are expected to emerge at a more advanced stage and have the worst prognosis in the following years. These patients represent an excess of mortality among cancer patients in the future.

In addition, many studies have shown an important delay in cancer treatment that requires appropriate changes in treatment decisions [47,48]. During the pandemic, oncologists’ hot topic was to ensure the continuum of care: oncologic departments were substantially reorganized in the management and maintenance of life-saving treatments.

The data included in this meta-analysis were obtained during the pre-vaccination phase of the SARS-CoV-2 pandemic. This scenario could be deeply upset by universal vaccination and immunity boosting. A recent European study supported novel evidence regarding the clinical efficacy against COVID-19 morbidity, mortality and sequelae of SARS-CoV-2 vaccines in a large real-world population of patients with cancer [49]. Despite new variants, such as Omicron, patients with cancer remain highly susceptible to SARS-CoV-2 if they are not vaccinated against SARS-CoV-2. The probable pathogenicity of different variants in patients with cancer remains an open question. Additionally, we were unable to explore the potential outcomes of the SARS-CoV-2 variants because this information was not available.

As a result of the available data, this systematic review has a number of limitations, including: (1) heterogeneity in the definition of COVID-19 severity; (2) retrospective nature of most studies (19 of 29); (3) variable follow-up times; (4) poor descriptions of control cohorts; and (5) no details on the cancer treatment, stage of disease, and histology of lung cancer. A large sample of the population with different geographies suggests that the SARS-CoV-2 pandemic has been managed differently in every nation in terms of prevention and control. Arayici et al. [39] suggested that different pandemic management and lifestyles can lead to increased or decreased susceptibility to cancer or SARS-CoV-2 infection and explained the heterogeneity of the collected data.

The studies included in this systematic review did not provide any details about COVID-19 variants. Most of the included papers refer to the analysis conducted in 2020, at which point B.1.1.7 (alpha), B.1.351 (beta), and B.1.617.2 (delta) variants were in circulation [50].

## 5. Conclusions

This systematic review and meta-analysis showed that lung cancer is an important comorbidity that modifies COVID-19 prognosis in terms of disease severity and mortality. This probably represents a risk factor for acquiring SARS-CoV-2 infection. This increased susceptibility could be explained by pathogenic mechanisms but also by the greater frequency of hospital access of oncological patients. Therefore, during the pandemic, it was necessary to find a compromise guaranteeing the continuum of care of cancer patients without exposing them to high risks. Thus, these data represent an important lesson for the recrudescence of the new pandemic.

An important step in unborn studies is to evaluate COVID- 19 mortality among vaccinated cancer patients to understand the contribution of mass vaccination campaigns to relieving this threat. It would be important to stratify patients according to the type of vaccine received, the number of doses received, and the time of vaccination because it could modify the antibody response.

## Figures and Tables

**Table 1:** Characteristics of included studies.

Author/Year	Type of Study	Country	N° Lung Cancer pts	Follow Up	Risk of COVID (HR, 95%CI)	Severe Disease	Mortality %	Quality	Bias (ROBINS)
Assad et al., 2020 [10]	Retrospective study	France	42	Median 25 days	NR	NR	↑	6	Low
Aveyard et al., 2021 [11]	Retrospective cohort study	UK	60	NR	NR	↑ ^*^	↑ ^*^	5	Low
Basse et al., 2020 [12]	Prospective study	France	18	28 days	NR	NR	↑ #	6	Low
Beltramo et al., 2020 [13]	Retrospective cohort study	France	977	NR	NR	= ^*^	↑ ^*^	5	Low
Benderra et al., 2021 [14]	Retrospective cohort study	France	85	30 days	NR	NR	↑ ^*^	6	Low
Bernard et al., 2021 [15]	Retrospective cohort study	France	873	NR	NR	NR	↑ ^*^	5	Low
Chen et al., 2022 [16]	Cohort study	USA	10,346	NR	NR	NR	↑ ^*^	5	Low
Dai et al., 2020 [17]	Retrospective multicenter study	China	31	53 days	NR	↑	↑	7	Low
De Joode et al., 2022 [18]	Prospective cohort study	Netherland	117	NR	NR	NR	↑ ^$^	6	Low
Farooque et al., 2021 [19]	Prospective study	Pakistan	159	At least 30 days	NR	NR	↑ ^*$^	6	Moderate
Fernandes et al., 2021 [20]	Retrospective cross-sectional study	Brazil	18	NR	NR	NR	↑ ^*$^	5	Low
Ferrari et al., 2022 [21]	Prospective study	Brazil	16	Median 61 days	NR	NR	↑ #	7	Low
Garassino et al., 2020 [22]	Cross-sectional and longitudinal cohort study	International	200	Median 15 days (IQR 8–24)	NR	NR	↑ ^**^	6	Low
Jee et al., 2020 [23]	Retrospective observational study	USA	29	NR	NR	↑ ^*^	NR	5	Low
Khusid et al., 2021 [24]	Retrospective cohort study	USA	14	NR	NR	= ^*^	↑ ^*^	5	Low
Kim et al., 2022 [25]	Retrospective study	USA	887	At least 30 days	NR	↑ ^*^	↑ ^*^	7	Low
Lee et al., 2020 [26]	Prospective observational study	UK	90	Max 39 days	NR	NR	=	7	Low
Lièvre et al., 2020 [27]	Retro-prospective cohort study	France	311	Median 34 days	NR	↑ ^*^	↑ ^*,°^	7	Low
Nader Marta et al., 2021 [28]	Retrospective, single-institute cohort study	Brazil	42	NR	NR	NR	↑ ^*$^	6	Low
Oh et al., 2021 [29]	Retrospective study	South Korea	769	NR	NR	NR	↑ ^*$^	6	Low
Ozdemir et al., 2021 [30]	Retrospective study	Turkey	157	Median 50 days (min 1- max 74)	NR	NR	↑ ^*$^	7	Low
Preda et al., 2022 [31]	Retrospective study	Romania	66	NR	NR	NR	Not significant	6	Low
Song et al., 2020 [32]	Retrospective multicenter study	China	61	NR	NR	NR	↑ ^$^	8	Low
Varnai et al., 2022 [33]	Prospective cohort study	UK	265	NR	NR	NR	↑ ^*$^	5	Low
Wang et al., 2020 [34]	Retrospective case-control study	USA	140	NR	↑ ^*^	NR	NR	5	Low
Wang et al., 2022 [35]	Retrospective cohort study	USA	2849	NR	↑	NR	NR	6	Low
Yang et al., 2021 [36]	Retrospective cohort study	South Korea	362	NR	↑ ^*^	↑ ^*^	↑ ^*^	4	Low
Yarza et al., 2020 [37]	Retro-prospective study	Spain	17	NR	NR	↑ ^*^	↑ ^*$^	6	Low
Zorzi et al., 2021 [38]	Retrospective population-based study	Italy	2256	NR	= ^*^	NR	↑ ^*^^	6	Low

* Multivariate analysis; ° compared with non-thoracic cancer; # compared to breast cancer; ^ compared to colorectal cancer; $ compared to other cancers; ** compared to small cell lung cancer, = similar, ↑ increased.

## Data Availability

Not applicable.
